# BMP-2 induces EMT and breast cancer stemness through Rb and CD44

**DOI:** 10.1038/cddiscovery.2017.39

**Published:** 2017-07-17

**Authors:** Peide Huang, Anan Chen, Weiyi He, Zhen Li, Guanglin Zhang, Zhong Liu, Ge Liu, Xueting Liu, Shuilian He, Gang Xiao, Feicheng Huang, Jan Stenvang, Nils Brünner, An Hong, Ju Wang

**Affiliations:** 1 Institute of Biomedicine, Guangdong Provincial Key Laboratory of Bioengineering Medicine, National Engineering Research Centre of Genetic Medicine, College of Life Science and Technology, Jinan University, Guangzhou 510632, China; 2 Section for Molecular Disease Biology, Department of Drug Design and Pharmacology, Faculty of Health and Medical Sciences, University of Copenhagen, 2200 Copenhagen N, Denmark; 3 Guangzhou Institutes of Biomedicine and Health, Chinese Academy of Sciences, 190 Kai Yuan Avenue, Science Park Luogang, Guangzhou 510530, China; 4 Department of Chemistry and Institute for Nano-Chemistry, Jinan University, Guangzhou 510632, China

## Abstract

Bone morphogenetic protein 2 (BMP-2) has been reported to facilitate epithelial-to-mesenchymal transition (EMT) and bone metastasis in breast cancer xenograft models. To investigate the role of BMP-2 in the development of breast cancer stem cells (BCSCs), and to further elucidate the mechanisms underlying its influence on breast cancer metastasis, we conducted a comprehensive molecular study using breast cancer cell lines and clinical samples. Our results showed that downregulation of Rb by BMP-2 was associated with ubiquitin-mediated degradation activated by phosphorylation of Rb via the PI3K/AKT signal pathway. In addition, the Smad signaling pathways are implicated in upregulation of CD44 protein expression by BMP-2. It was suggested that cross-talk exists between Rb and CD44 signaling pathways, as recombinant human BMP-2 (rhBMP-2) was found to regulate CD44 expression partly through Rb signals. In clinical tissues, BMP-2 was positively and negatively correlated with CD44 and Rb expression, respectively. Based on the *in vitro* and *in vivo* results, we have established an integrated mechanism by which rhBMP-2 induces EMT and stemness of breast cancer cells via the Rb and CD44 signaling pathways, which then contribute to breast cancer metastasis. These findings may be helpful for developing new strategies for the treatment and prognosis of advanced breast cancer.

## Introduction

Breast cancer is a leading cause of cancer deaths among women worldwide, second only to lung cancer;^1^ metastasis is the main cause for breast cancer related deaths.^2,3^

The concept that cancer stem cells (CSCs) drive cancer formation and progression has recently gained attention. Studies have shown that breast cancer stem cells (BCSCs, marked as CD44^+^/CD24^−^) promote tumor progression and exhibit enhanced invasive properties to favor distant metastasis in patients.^4,5^

Bone morphogenetic proteins (BMPs) are multifunctional growth factors belonging to the TGF-*β* superfamily. BMP-2 was reported to facilitate epithelial-to-mesenchymal transition (EMT)^6^ and promote the motility and invasiveness of breast cancer cells *in vitro* and in mouse xenograft model.^7,8^ A recent study reported that the BMP-2 pathway can be activated by pollutants exposure, and contributes to stem cell transformation and breast cancer initiation.^9^ However, the mechanisms by which BMP-2 promotes EMT and breast cancer metastasis, and its relationship with BCSC development, remain largely unknown.

Although EMT is a well-characterized process during normal development, its role in cancer progression is controversial.^10^ Many studies suggested that EMT occurs during the generation of cancer stem cells within primary tumors capable of metastasis.^11,12,13^ However, in some cases, a partial EMT or MET (mesenchymal–epithelial transition) is necessary, such as during differentiation and formation of tubules in kidney development.^14,15^

Rb (retinoblastoma) is a well-known cancer suppressor that initiates and maintains cell cycle arrest, modulates apoptosis, and is essential for early embryonic development. Rb regulates cell growth and differentiation by modulating the activity of transcription factors such as E2F family members.^16,17^ Inactivation of Rb in the mouse mammary epithelium induces aggressive and metastatic mammary tumors with basal stem cell-like phenotypes.^18^

CD44, an alternatively spliced transmembrane protein, functions as a receptor for hyaluronan, and act as the co-receptor for multiple receptor kinases linked with breast cancer.^19^ CD44 expression is essential for maintenance of the cancer stem cell phenotype.^20^

In this study, we investigated the role of BMP-2 in BCSC development. We aimed to elucidate the mechanisms underlying the influence of BMP-2 on breast cancer progression using recombinant human BMP-2 (rhBMP-2). This is the first study that reveals an integrated mechanism behind the effect of BMP-2 on cancer stem cell formation and breast cancer metastasis.

## Results

### rhBMP-2 induced EMT-like transformation, enhanced the migration/invasion ability of breast cancer cells *in vitro*, and promoted lung metastases of breast cancer *in vivo*

**Figure 1:**
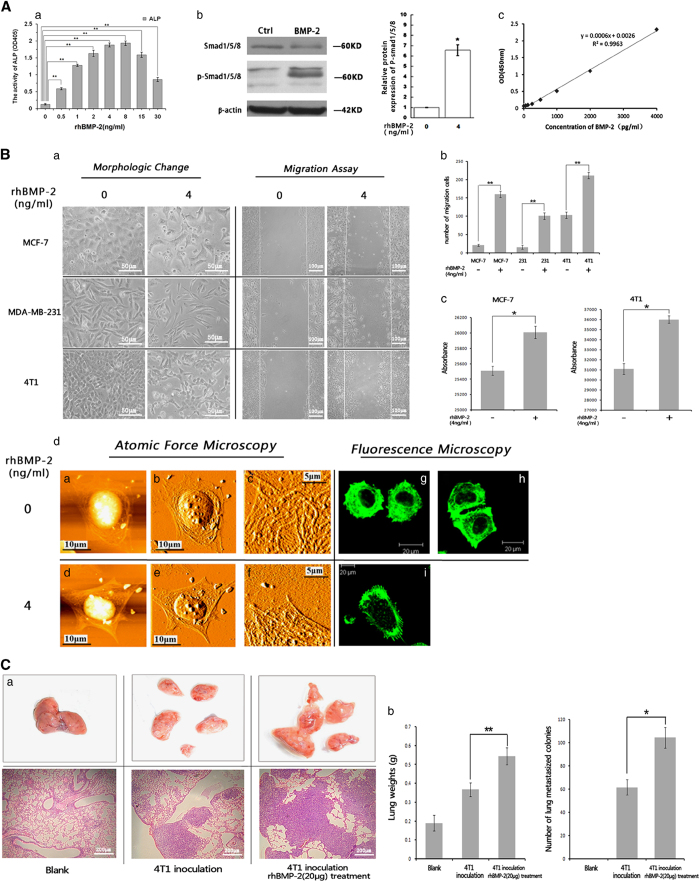
rhBMP-2 induced EMT-like transformation, enhanced migration/invasion ability of breast cancer cells *in vitro*, and promoted lung metastases of breast cancer cells *in vivo*. (**A**) Activity assay of rhBMP-2. (a) C2C12 cells were induced with different concentrations of rhBMP-2(0, 0.5, 1, 4, 8, 16, 32 ng/ml) for 72 h, and ALP activity was detected by using the ALP substrate kit (Bio-Rad, USA). Data are displayed as means±S.D. (**P<0.01, *n*=3). (b) MCF-7 cells were induced with 4 ng/ml rhBMP-2 for 24 h, and Smad1/5/8 and p-Smad1/5/8 were detected by western blot (*P<0.05, *n*=3). (c) ELISA was performed to detect the expression level of BMP-2 in MCF-7 cells, and canonical plotting was carried out. (**B**) rhBMP-2 induced EMT-like transformation and enhanced migration/invasion ability of breast cancer cells. (a–b) Human breast cancer cell lines MCF-7 and MDA-MB-231, and a mouse breast cancer cell line 4T1, were induced with 4 ng/ml rhBMP-2 for 24 h. Morphological changes and the numbers of migrating cells were determined using an inverted microscope (Olympus ix71). Data are shown as mean±S.D. (**P<0.01, *n*=3). (c) MCF-7 and 4T1 cells were inoculated into the upper surface of QCMTM 96-well plates, and were induced with 4 ng/ml rhBMP-2 for 24 h. Cells invading through the membrane were stained and measured under 480/520-nm transmitted light. Values are presented as mean±S.D. (*P<0.05, *n*=3). (d) MCF-7 cells were induced with 4 ng/ml rhBMP-2 for 24 h, and the actin cytoskeletons were observed under an atomic force microscope (a–f) or a fluorescence microscope (g–i). (**C**) 60 BALB/c female mice were randomly divided into three groups (*n*=20). Experimental groups were intravenously injected with 4T1 cells, whereas the un-injected group was set as blank control. Mice in one of the experimental groups were treated daily with 20 *μ*g rhBMP-2 via the tail vein for 21 days. The other two groups were treated with PBS for 21 days. Animals were killed, and lung weight and the number of tumor nodules were measured. (a) Photos of lung lobe (upper panels) and H&E staining of lung biopsy (lower panels) from control, 4T1 inoculated, and 4T1 inoculated rhBMP-2-treated mice. (b) Left: average weight of lungs from control, 4T1 inoculated, and 4T1 inoculated rhBMP-2-treated mice. Values are presented as mean±S.D. (**P<0.01, *n*=20). Right: average number of lung metastasized colonies from control, 4T1 inoculated, and 4T1 inoculated rhBMP-2-treated mice. Data are presented as mean±S.D., (*P<0.05, *n*=3).

To evaluate the activity of rhBMP-2 (R&D Systems, Minneapolis, MN, USA), alkaline phosphatase (ALP) was used as a marker for detecting rhBMP-2 activity. Various concentrations of rhBMP-2 were used to induce the differentiation of mouse myoblast C2C12 cells into osteoblasts. Results showed that ALP activity reached the maximum value at rhBMP-2 concentrations of 4–8 ng/ml (Figure 1Aa) (**P<0.01, *n*=3). Therefore, 4 ng/ml was chosen as the optimal dose of rhBMP-2 used to induce MCF-7 cells in subsequent experiments. Results indicated that 4 ng/ml rhBMP-2 was able to activate Smad-dependent signaling pathways in MCF-7 cells (Figure 1Ab).

BMP-2 level (compared with total cell protein) in MCF-7 cells was measured via ELISA. Intracellular concentration of BMP-2 was found to be 356.46 pg/mg, whereas extracellular BMP-2 concentration was 499.59 pg/mg (Figure 1Ac, Supplementary Table S1). This showed that native BMP-2 protein was produced in MCF-7 breast cancer cells at relatively low levels.

To investigate the potential role of BMP-2 in breast cancer development, we treated three breast cancer cell lines (MCF-7, MDA-MB-231, and a mouse breast cancer cell line 4T1) with rhBMP-2 for 24 h. We found that rhBMP-2 induced elongated morphologic changes in all three breast cancer cell lines. Especially in MCF-7 cell line, rhBMP-2 drove MCF-7 cells from a paving stone sheet-like shape to fibroblastic-spindle shape with pseudopodia formation (Figure 1Aa), which was considered to be a characteristic of EMT.^21^ This indicated that rhBMP-2 may promote EMT of breast cancer cells. As actin cytoskeleton reorganization is considered another characteristic of EMT, we then used atomic force microscopy (AFM) and fluorescence microscopy to observe the structure of actin cytoskeleton in rhBMP-2-induced MCF-7 cells. We found that rhBMP-2 promoted reorganization of the actin cytoskeleton in MCF-7 cells (Figure 1Ad).

We then performed a wound-healing assay on rhBMP-2-induced breast cancer cells and found that 4 ng/ml rhBMP-2 significantly enhanced breast cancer cell migration into the wound (Figure 1Aa–b). In addition, results from transwell invasion assays demonstrated that the average numbers of rhBMP-2-treated MCF-7 and 4T1 cells invading through the membrane were significantly increased compared to those in the control group (Figure 1Ac). Therefore, rhBMP-2 treatment could enhance both migration and invasion capabilities of breast cancer cells *in vitro*.

To further investigate the effect of rhBMP-2 on breast cancer *in vivo*, we generated a lung metastatic breast cancer model by inoculating Balb/c mice with 4T1 murine breast cancer cells via the caudal vein. As shown in Figure 1C, the number of metastatic tumor nodes in the lung was significantly higher in mice treated with rhBMP-2 than in those treated with PBS. This suggests that rhBMP-2 promotes breast cancer metastasis *in vivo*.

### rhBMP-2 induced differential expression of the genes associated with tumor metastasis in MCF-7 breast cancer cells

To determine the molecular mechanisms underlying the effects of BMP-2 on breast cancer cells, we used the RT^2^ *Profiler* PCR Array (Qiagen, Hilden, Germany) to detect changes in the expression of 84 genes known to be associated with tumor metastasis (Supplementary Table S2).

**Figure 2:**
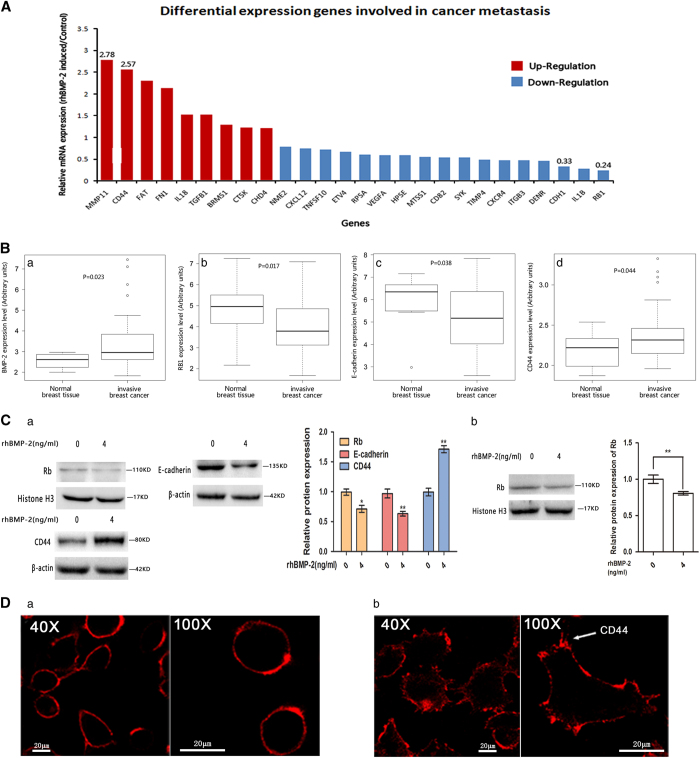
rhBMP-2 induced differential mRNA expression of genes involved in tumor metastasis, and affected the expression and distribution of proteins in breast cancer cells. (**A**) MCF-7 cells were induced with 4 ng/ml rhBMP-2 for 24 h, and the expression changes of 84 genes associated with tumor metastasis were detected by Human Tumor Metastasis RT^2^ Profiler PCR Array. The 26 significantly upregulated (fold difference>1.2; *P*-value<0.05; indicated by the red bar) and downregulated genes (fold difference<0.8; *P*-value<0.05; indicated by the blue bar) was shown. (**B**) Gene expression levels of (a) *BMP-2*, (b) *RB1*, (c) *E-cadherin* and (d) *CD44* in invasive breast cancer and normal breast tissues from the GSE10797 breast cancer data set; differences were assessed by the unpaired-Student’s *t*-test. (**C**) (a) MCF-7 cells were induced with 4 ng/ml rhBMP-2 for 24 h; protein levels of Rb, E-cadherin, and CD44 were determined by western blot. Data are presented as mean±S.D., (**P<0.01, *n*=3). (b) MDA-MB-231 cells were induced with 4 ng/ml rhBMP-2 for 24 h; protein levels of Rb were determined by western blot. Data are presented as mean±S.D., (**P<0.01, *n*=3). (**D**) Distribution of the CD44 protein (arrow indicates CD44 protein) in control (a; 0 ng/ml rhBMP-2, 24 h) and rhBMP-2-induced (b; 4 ng/ml rhBMP-2, 24 h) MCF-7 cells.

Differential expressions of the 84 genes in rhBMP-2-induced MCF-7 and control cells were calculated (Supplementary Table S3). We identified 26 genes that were either significantly upregulated (Fold difference>1.2; *P*-value<0.05) or downregulated (Fold difference<0.8; *P*-value<0.05) (Figure 2A). The most upregulated genes were *CD44* and *MMP11*, whereas the most downregulated genes were *RB1* and *CDH1* (E-cadherin). To further investigate the relationship between the expression status of these four genes and the metastatic phenotype of clinical breast cancer, we analyzed the expression of these four genes in a data set (available online, GSE10797) consisting of normal breast tissues and invasive breast cancer samples. We found that expressions of *BMP-2* and *CD44* were significantly upregulated, whereas expressions of *RB1* and *CDH1* (E-cadherin) were significantly downregulated in invasive breast cancer (Figure 2B). This suggested that the expression of these four genes is associated with invasiveness of breast cancer cells.

Western blot was performed to determine whether rhBMP-2 could affect protein expressions of Rb, E-cadherin, and CD44. Rb, and E-cadherin were downregulated in MCF-7 cells; CD44 was upregulated following rhBMP-2 induction (Figure 2Aa). Similarly, in MDA-MB-231 cells, which were considered more aggressive than MCF-7 cells, Rb expression was downregulated upon rhBMP-2 induction (Figure 2Ab). As the 4T1 cell line was derived from rodents, we did not perform the assay on these cells.

CD44 is a cell-surface glycoprotein involved in cell–cell interactions, cell adhesion, and migration.^22^ Immunocytochemistry assays demonstrated that rhBMP-2 upregulated CD44 expression and induced the redistribution of cellular CD44 to the leading edges and lamellipodia of MCF-7 cells (Figure 2D).

### rhBMP-2 promoted Rb phosphorylation and degradation through Smad-independent PI3K/AKT signaling pathways, and modulated the migration ability of MCF-7 breast cancer cells

**Figure 3:**
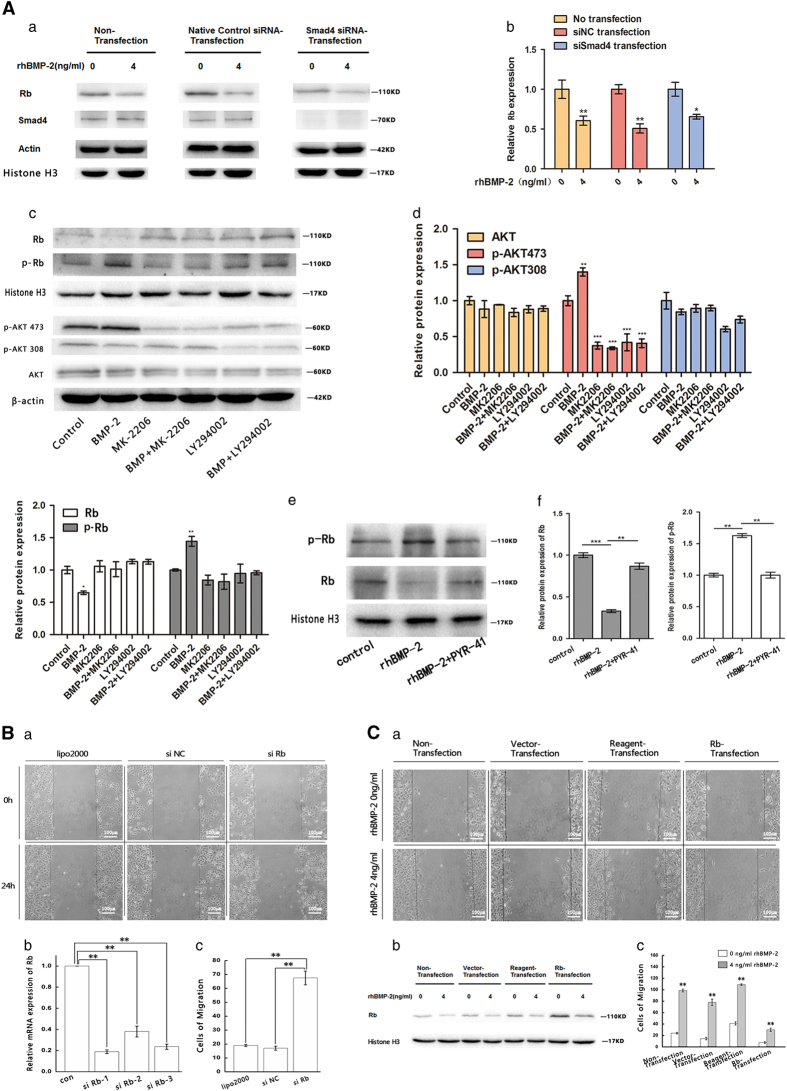
rhBMP-2 promoted Rb phosphorylation and degradation through Smad-independent PI3K/AKT signaling pathway, and affected the migration ability of MCF-7 breast cancer cells. (**A**) (a, b) MCF-7 cells transiently transfected with native control siRNA (siNC) or Smad4 siRNA for 24 h. Transfected and non-transfected MCF-7 cells were induced with 4 ng/ml rhBMP-2 for 24 h, and expression levels of Smad4 and Rb were determined by western blot. Data are presented as mean±S.D. (**P<0.01, *P<0.05, *n*=3). (c, d) PI3K and AKT inhibitors, LY294002 and MK-2206, were added to MCF-7 cells 1 h before rhBMP-2 induction. MCF-7 cells were induced with 4 ng/ml rhBMP-2 for 24 h. Relative protein levels were analyzed by western blot. Data are presented as mean±S.D. (***P<0.005, **P<0.01, *P<0.05, *n*=3). (e, f) Ubiquitin-activating enzyme E1 inhibitor, PYR-41, was incubated with MCF-7 cells for 30 min prior to the 24- h rhBMP-2 (4 ng/ml) induction. Rb and phosphorylated Rb were analyzed by western blot; data are presented as means±S.D. (***P<0.005, **P<0.01, *P<0.05, *n*=3). (**B**) (a) Wound-healing assay was performed to evaluate the migration of MCF-7 cells transiently transfected with siRb, negative control siRNA (siNC), or the transfection reagent (lipo2000) for 24 h. (b) Real-time PCR was performed to examine the targeting efficiency of Rb stealth RNAi (siRNA). The most effective siRNA siRb-1 was chosen for the following experiments. Values presented as mean±S.D., (**P<0.01, *n*=3). (c) Migration abilities of MCF-7 cells transiently transfected with siRb-1, negative control siRNA, or the transfection reagent (lipo2000). The average number of cells migrated into the wound was shown for each group. Values are presented as mean±S.D. (**P<0.01, *n*=3). (**C**) (a) pcDNA3.1-Rb over-expression plasmids were constructed using an Endo-free plasmid mini kit I. MCF-7 cells were transfected with the pcDNA3.1 vector, the transfection reagent, or the pcDNA3.1-Rb plasmids at 37 °C for 24 h. Cells were induced with 4 ng/ml rhBMP-2 for another 24 h, and wound-healing assay was performed to detect variations in migration of MCF-7 cells. (b) Rb protein levels in non-transfected control, vector-transfected, reagent-transfected, and Rb plasmid transfected groups, as analyzed by western blot. (c) Numbers of migratory cells in non-transfection control, vector-transfected, reagent-transfected, and Rb plasmid transfected groups. Values are expressed as mean±S.D. (**P<0.01, *n*=3).

To explore the role of Smad-dependent signal pathways in association with BMP-2 and Rb, we silenced Smad4 in MCF-7 cells by specific Smad4 siRNA. We found that silencing of the Smad4 protein in MCF-7 cells did not affect rhBMP-2-induced downregulation of Rb (Figure 3Aab).

We then examined Smad-independent signal pathways, especially the PI3K/Akt signaling pathway. We found that rhBMP-2 significantly increased the phosphorylation of Akt (473) and Rb (Ser807/811), and downregulated the expression of Rb in MCF-7 cells (Figure 3Aab); no effect on AKT (308) was observed. Specific inhibitors of PI3K (LY294002) and Akt (MK-2206) were used to study the role of PI3K/Akt signaling on rhBMP-2-induced changes in Rb expression. Results indicated that phosphorylation of Akt (473) and Rb (Ser807/811) was significantly decreased when cells were pre-treated with LY294002 or MK-2206. Concomitant impairments in Rb downregulation were also observed (Figure 3Aab).

Phosphorylation is known to trigger ubiquitination and degradation of regulatory proteins in many important cell signaling processes.^23^ Therefore, we used a cell permeable inhibitor of ubiquitin-activating enzyme E1, PYR-41,^24^ to study the phosphorylation and potential degradation of Rb (Figure 3A e). We found that downregulation of Rb induced by rhBMP-2 was significantly impaired when cells were pre-treated with PYR-41. This was correlated with reduced phosphorylation in Rb (Ser807/811) (Figure 3Af). This strongly suggested that phosphorylation of Ser807/811 sites triggers the ubiquitination and subsequent degradation of Rb. Our results suggested that BMP-2 induces phosphorylation and ubiquitin-dependent degradation of the Rb protein through a Smad-independent PI3K/Akt/Rb signaling pathway.

To gain further insights into the relationship between Rb protein levels and the migration capability of MCF-7 breast cancer cells, we used specific siRNA to deplete Rb proteins in MCF-7 cells. In addition, we constructed a pcDNA3.1-Rb over-expression plasmid, which was used to transiently transfected MCF-7 cells. A wound-healing assay was subsequently carried out to determine changes in MCF-7 cell motility. We found that depletion and over-expression of the Rb protein significantly enhanced and weakened the motility of MCF-7 cells, respectively (Figures 3B and C).

### rhBMP-2 upregulated CD44 protein expression and promoted cell motility and BCSC development via Smad-dependent and PI3K/Akt-Rb signaling pathways

**Figure 4:**
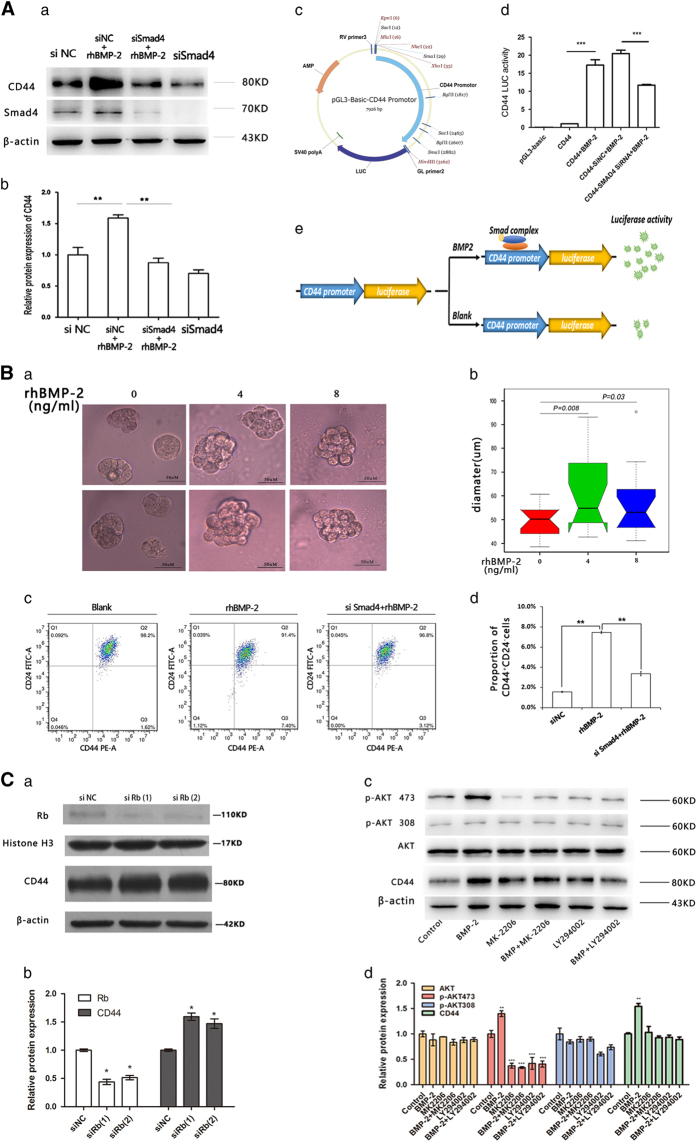
rhBMP-2 upregulated CD44 protein expression, and promoted cell motility and BCSC development via the Smad and PI3K/Akt-Rb signaling pathway. (**A**) (a, b) MCF-7 cells were transiently transfected with native control siRNA (siNC) or Smad4 siRNA for 24 h, after which they were induced with 4 ng/ml rhBMP-2 for 24 h. Smad4 and CD44 protein expressions were analyzed by western blot. Values are expressed as mean±S.D. (**P<0.01, *P<0.05, *n*=3). (c, d). CD44 reporter plasmid was constructed by adding the CD44 promoter sequence (3135 bp) into a pGL3-basic luciferase reporter vector. MCF-7 cells were transfected with the pGL3-basic control plasmid, the CD44 reporter plasmid, the *Renilla* luciferase expression construct, siNC, or siSmad4 for 24 h. The CD44 reporter, CD44 reporter plus siNC, and CD44 reporter plus siSmad4 transfected cells were induced by 4 ng/ml rhBMP-2 for 24 h. Activities of CD44 were measured by dual luciferase assay (Promega). Values are expressed as mean±S.D., (***P<0.005, *n*=3). (e) Schematic diagram showing rhBMP-2-induced upregulation of CD44 via binding of Smad4 to the promoter of CD44. (**B**) (a) MCF-7 cells (10^3^ cells/ml) were mixed with 5% Matrigel medium at a 1:1 ratio, and were inoculated into 24-well plates. Cells were induced by different concentrations of rhBMP-2 (0, 4, 8 ng). All cell cultures were incubated at 37 °C and 5% CO_2_ for 3 days. Tumor spheres were observed under an inverted microscope. Representative photos of MCF-7 tumor spheres from the three groups are shown. (b) Diameters of 30 randomly chosen tumor spheres in each group are displayed in boxplots (*n*=30, differences between groups were assessed by the Student’s *t*-test). (c, d). MCF-7 cells were divided into three groups (blank control, rhBMP-2-induced, siSmad4 rhBMP-2-induced). Cells in the siSmad4 rhBMP-2-induced group were transiently transfected with Smad4 siRNA for 24 h. Cells in both the rhBMP-2-induced group and siSmad4 rhBMP-2-induced groups were induced with 4 ng/ml rhBMP-2 for 24 h. The CD44+/CD24- cell population in each group was detected via flow cytometry. Data are expressed as mean±S.D., (**P<0.01, *n*=3). (**C**) (a) Expressions of Rb and CD44 in MCF-7 cells were evaluated by Western blot following siRNA-mediated Rb silencing. (b) Relative expression levels of Rb and CD44 in normal (NC) and Rb interfered (siRb1, siRb2) cells. Values are expressed as mean±S.D., (*P<0.05, *n*=3). (c–d) PI3K and AKT inhibitors, LY294002 and MK-2206, were added to MCF-7 cells 1 h prior to rhBMP-2 induction. MCF-7 cells were then induced with 4 ng/ml rhBMP-2 for 24 h (this experiment was the same as Figure 3Ac and the same lysates were used to detected protein levels of Rb, p-Rb, AKT, p-AKT, *β*-actin, Histone3, as well as CD44 by western blot). Relative protein expressions were determined by western blot. Values are expressed as mean±S.D. (**P<0.01, *P<0.05, *n*=3).

Similarly, we found that depletion of CD44 by si-CD44 significantly decreased the motility of MCF-7 cells, and neutralized the stimulatory effect of rhBMP-2 (Supplementary Figures 1A–C). To investigate the relationship between rhBMP-2 and CD44, we silenced Smad4 in MCF-7 cells with 4 ng/ml rhBMP-2 for 24 h, and found that upregulation of CD44 by rhBMP-2 was inhibited (Figure 4Aab). Upregulation of the CD44 protein may possibly be mediated by the classical Smad signaling pathway. The Smad4 protein possesses a MH1 domain, which recognizes the double-stranded DNA sequence motif 5′- CAGAC-3′ known as the Smad-binding element (SBE).^25^ The promoter sequence of CD44 was found to harbor an SBE-rich region (−3090 to −2705 bp, Supplementary Table S4). We hypothesized that the Smad complex may bind to these SBEs and regulate expression of CD44. We therefore constructed a CD44 luciferase reporter system (Figure 4Ac), and found that rhBMP-2 markedly enhanced CD44 promoter activity in MCF-7 cells, which was impaired by interfering Smad4 expression (Figure 4Ad). This result demonstrated again that the Smads complex may bind to the CD44 promoter and directly regulate rhBMP-2-mediated increase in CD44 expression (Figure 4Ae).

To investigate whether stemness is enhanced following BMP-2 treatment, we quantified CD44^+^/CD24^−^ populations in rhBMP-2 (4 ng/ml)-induced MCF-7 cells using the FACSCalibur flow cytometer. We found that the CD44^+^/CD24− cells induced by rhBMP-2 showed a 4.8-fold increase compared with untreated cells (Figure 4A a b). We also performed 3D tumor spheroid formation assays to evaluate the effect of rhBMP-2 on proliferation of BCSCs. We observed an increase in the average size of tumor spheroids in the rhBMP-2 treatment group compared with in the control group (Figure 4Aab). These results demonstrated that rhBMP-2 promotes the development of CSCs in MCF-7 cells. In addition, siSmad4 neutralized the rhBMP-2-induced upregulation of CD44^+^/CD24^−^ population in MCF-7 cells (Figure 4Aab), which demonstrated that the stimulatory effect of rhBMP-2 on BCSC development was mediated through the Smad signaling pathway.

To determine the relationship between Rb and CD44, we assessed the level of CD44 in Rb- depleted MCF-7 cells by western blot. Our results showed that CD44 expression was significantly upregulated in Rb-silenced cells compared with that in control MCF-7 cells (Figure 4Aab). Further study showed that CD44 upregulation by rhBMP-2 was impaired when cells were pre-treated with PI3K and AKT inhibitors (LY294002 and MK-2206) (Figure 4Aab). These results suggested that expression of CD44 was partially regulated by PI3K/Akt-Rb signaling.

### BMP-2 expression was inversely correlated with Rb expression, and was positively correlated with CD44 expression in breast carcinoma tissues

To determine the protein levels of BMP-2, Rb, and CD44 in breast cancer tissues, we collected 40 breast carcinoma tissue samples (classification of the samples is shown in Supplementary Table S5), and immunohistochemical analysis was performed.

The widely accepted German semi-quantitative scoring system used to quantify staining intensity and area was used.^26^ Breast tumor samples were divided into three categories based on BMP-2 levels, and were defined by their expression scores as low (0–4), medium (5–8), or high (9–12).

**Figure 5:**
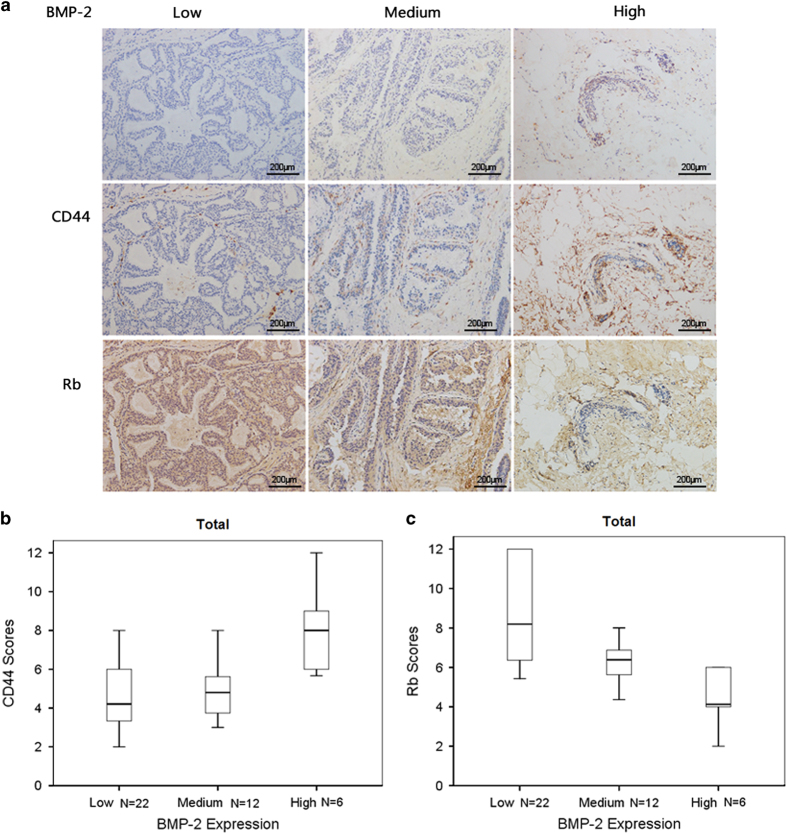
BMP-2 expression was inversely correlated with Rb expression and positively correlated with CD44 expression in the breast carcinoma cohort. (**a**) Immunohistochemistry was performed to detect expressions of BMP-2, Rb, and CD44 in 40 breast cancer tissue samples. Samples were categorized into three groups based BMP-2 levels defined by their expression scores: low (0–4), medium (5–8), and high (9–12). Representative images show the expression of Rb and CD44 in samples belonging to the low, medium, and high BMP-2 groups. (**b**) Plot demonstrating positive correlation between BMP-2 and CD44 expression in our breast carcinoma cohort. CD44 expression in each group was also defined by their scores. The asterisks represent extreme outliers, whereas the dots stand for mild outliers. (**c**) Plot demonstrating negative correlation between BMP-2 and Rb expression in our breast carcinoma cohort. Rb expression in each group was also defined by their scores. The asterisks represent extreme outliers, whereas the dots stand for mild outliers.

By analyzing the immunohistochemical images and immunoreactive scores of BMP-2, CD44, and Rb proteins in each sample, we confirmed that BMP-2 expression was positively correlated with CD44 expression in the breast carcinoma cohort (Figures 5a and b, Supplementary Figure 2). In contrast, BMP-2 expression was found to be negatively correlated with Rb expression (Figure 5a and c; Supplementary Figure 3).

## Discussion

BMPs are highly conservative functional proteins belonging to the transformation growth TGF-*β* superfamily. They were originally identified based on their ability to facilitate bone formation at extra-skeletal sites. However, for the last decade, they have been extensively studied in several cancers.^27,28,29,30^ Functional studies revealed contradictory roles of BMPs in both cancer promotion and inhibition.^31,32,33,34^ Especially in breast cancer, different BMP ligands have been shown to decrease as well as increase cancer cell growth and migration.^35^ In our previous study, we showed that BMP-2 inhibited cancer cell growth both in *vitro* and in *vivo* by inducing G1 arrest and apoptosis in MDA-MB-231 and MCF-7 human breast cancer cell lines.^36^ Here, we further investigated the mechanism underlying the influence of BMP-2 on breast cancer metastasis through a comprehensive molecular study using breast cancer cell lines and clinical breast cancer samples. Our results indicated that BMP-2 promoted EMT and migration/invasion of breast cancer cells. In addition, it was shown to favor lung metastasis *in vivo*, which was in agreement with several other recent studies.^6,7,8^ A possible interpretation to the dual roles of BMP-2 is that in the early stages of breast cancer development, BMP-2-induced apoptosis plays a dominant role owing to low number of tumor cells; as the tumor continues to develop, EMT and stemness induced by rhBMP-2 have more significant roles than programmed tumor cell deaths. Therefore, the timing of BMP-2 induction may lead to differential outcomes in tumors, such as death or metastasis.

Notably, rhBMP-2 promoted the formation of tumor spheroids and increased the population of CD44^+^/CD24^−^ cells in MCF-7 breast cancer cells (Figure 4B) in this study. These observations suggest that rhBMP-2 enhances the stemness of breast cancer cells. BMP signaling was thought to have important roles in regulating the survival and maintenance of CSCs,^37,38^ and the contributions of BMP-2 to the development of CSCs in different cancers are debated.^34,39,40,41,42^ Although BMP-2 was shown to inhibit CSC tumorigenicity in osteosarcoma and renal cell carcinoma,^34,39^ in support of our finding, BMP-2 was also reported to promote CSC formation in colon and ovarian cancers.^41,42^ Interestingly, in another study, BMP-2/7 heterodimer was shown to inhibit CSC subpopulations and onset of bone metastases.^40^ Therefore, the relationship between BMP signaling and CSC remains controversial. One possible explanation may be that the BMP-2/7 heterodimer introduces a competitive effect on other BMP signals, thus disrupting regulatory processes of CSC formation and metastases. Nevertheless, the contradictory reports also unveiled the complexities behind the signaling mechanisms of BMP-2 in EMT and CSC formation. As a result, there is an imperative need to further study the signaling mechanisms underlying the effect of BMP-2 on EMT and BCSC formation.

We investigated the mRNA and protein expressions of 84 genes known to be involved in tumor metastasis in rhBMP-2-treated MCF-7 cells; rhBMP-2 significantly downregulated Rb and E-cadherin and upregulated CD44 expression in MCF-7 cells (Figure 2). Rb is a well-known tumor suppressor. It inhibits transcription of genes required for the G1-S phase, which results in cell cycle arrest.^16^ It is usually inactivated by mutations in several human cancers.^43,44^ Furthermore, functional loss of the Rb gene has been shown to contribute to aggressive tumor phenotypes and induce EMT in breast cancer.^18,21,45^ Arima *et al.*^21^ showed that Rb can bind to the E-cadherin promoter in conjunction with AP-2*α*. Knockdown of Rb by small interfering RNA in MCF-7 breast cancer cells resulted in deregulation of E-cadherin, disruption of cell–cell adhesion, and induction of mesenchymal-like phenotypes. Our study further showed that deregulation of Rb, E-cadherin, and disruption of cell–cell adhesion was induced by rhBMP-2 in MCF-7 cells. These findings suggested that E-cadherin is downregulated by rhBMP-2 via depletion of Rb, and contributes to EMT. Our study highlighted the crucial role of Rb in BMP-2 signaling during EMT induction.

We also found that BMP-2 downregulated Rb protein through a Smad-independent PI3K/AKT signaling pathway in breast cancer cells. Results indicated that Rb was phosphorylated and subjected to ubiquitin-dependent degradation, which was mediated by the PI3K/Akt signaling pathway (Figure 3A a–f). Consistent with our findings, phosphorylation has been shown to be a common strategy that triggers the ubiquitination and degradation of certain proteins in many important cell processes.^23,46^ Different to our finding of phosphorylation of Rb on Ser807/811, phosphorylation of Rb on Ser567 had been reported to trigger ubiquitin-dependent degradation of Rb in melanoma cell though it was activated by p-38 signal pathways, and resulted in cell apoptosis.^47^ Moreover, the PI3K/Akt signaling pathway has been shown to activate phosphorylation of Rb, leading to disassociation of the Rb-E2F complex and cell proliferation but they only speculated the degradation of Rb caused by proteasome-mediated degration,^47,48,49^ so we firstly demonstrated this mechanism to down-regulate Rb by rhBMP-2 to promote metastasis in MCF-7 cells.

CD44 is a transmembrane glycoprotein widely expressed in physiological and pathological systems,^22^ and is associated with tumor metastasis and BCSCs.^4,5,50^ According to recent studies, the cleaved intracellular domain of CD44 (CD44ICD) activates stemness factors such as Nanog, Sox2, and Oct4, and contributes to tumorigenesis of breast cancer, which may explain the effect of CD44 on BCSC maintenance.^51^ In this study, we showed that BMP-2 upregulated CD44 expression and promoted CSC development in breast cancer cells. On one hand, our results showed that rhBMP-2 caused CD44 to localize in the leading edges and lamellipodia of MCF-7 cells (Figure 2D), which was consistent with Kim *et al*.^52^ Their study showed that the formation of F-actin-positive cellular protrusions such as filopodia and invadopodia, which are essential for initiation and progression of metastasis,^53^ was CD44-dependent. On the other hand, rhBMP-2 upregulated the expression of CD44 proteins and promoted the stemness of breast cancer cells (Figure 4B). These findings indicate that CD44 enables breast cancer cells to disseminate from the primary tumor, and promotes their ability to self-renew and colonize to distant sites.

We then sought to further decipher the signaling pathways between BMP-2 and CD44. Smad proteins are the central mediators of the TGF-*β* family of signaling molecules. Although the R-Smads, Smad1/5/8, are phosphorylated following activation by BMP receptors, the Co-Smad, Smad4, are recruited and combined with p-Smad1/5/8; the fully assembled complex then acts as a transcription factor to regulate the expression of target genes.

Through Smad4 siRNA silencing and the CD44 promoter luciferase reporter system, we found that rhBMP-2 upregulated CD44 expression in MCF-7 cells via binding of Smad4 to the promoter sequence of CD44 (Figure 4Aa–e), which contains a SBE-rich region (Supplementary Table S4).

Our study further showed that upregulation of CD44 was partially caused by activation of the PI3K/Akt pathway and reduction in Rb protein level (Figure 4C). This result was consistent with that of a recent study, which showed that CD44 expression was required for collective motility and metastatic progression initiated by loss of Rb function in breast cancer.^52^ This suggested that cross-talks between Rb and CD44 pathways are required for BMP-2-dependent EMT and development of BCSCs.

In support of this conclusion, our immunohistochemical assays performed in clinical breast cancer samples also shown an inverse correlation between the expression levels of BMP-2 and Rb and a positive correlation between BMP-2 and CD44 (Figures 5a–c).

**Figure 6:**
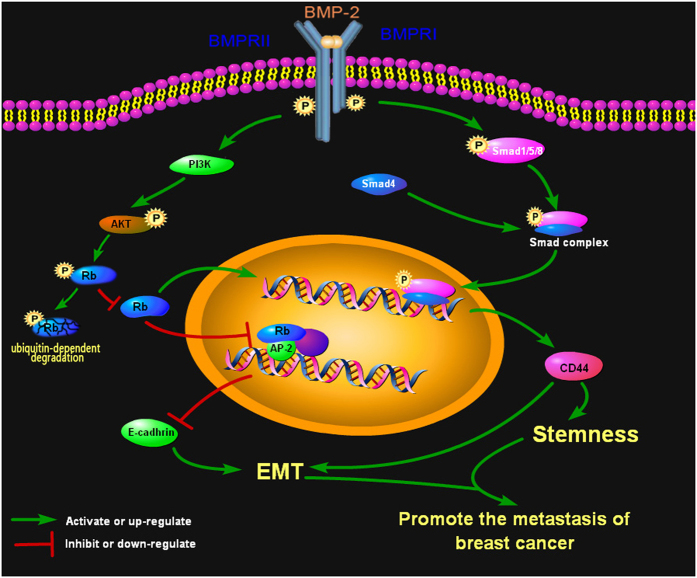
BMP-2 induced EMT and breast cancer stemness through Rb and CD44 signaling. This diagram depicts the signaling pathway by which rhBMP-2 is found to induce EMT and stemness of breast cancer cells through Rb and CD44 to contribute to breast cancer metastasis. PI3K/AKT and Smad signaling pathway are implicated in the regulation of Rb and CD44 by rhBMP-2; cross-talk exists between Rb and CD44 signaling pathways.

In summary, this is the first study demonstrating that BMP-2 promotes EMT and breast cancer stemness via Rb and CD44 signaling pathways (Figure 6). We found that Rb and CD44 are two key mediators in the rhBMP-2 signaling pathway. In addition, both PI3K/AKT and Smad signaling are implicated in the regulation of Rb and CD44 expression. Our *in vitro* and *in vivo* findings highlight the crucial roles of BMP-2, Rb, and CD44 in breast cancer metastasis, which may provide new strategies for the treatment and prognosis of advanced breast cancer.

## Materials and methods

### Cell lines, cultures, and treatments

The human breast cancer cell lines MCF-7 and MDA-MB-231, mouse myoblasts cell line C2C12, and mouse mammary carcinoma cell line 4T1 were obtained from the American Type Culture Collection (ATCC, USA). MCF-7 and C2C12 cells were cultured in Dulbecco’s modified Eagle’s medium (DMEM) (Gibco, ThermoFisher Scientific, Waltham, MA, USA) supplemented with 10% fetal bovine serum (FBS) (Gibco). MDA-MB-231 cells and 4T1 cells were cultured in RPMI 1640 (Gibco) with 10% FBS. All cell lines were grown in 5% CO_2_ at 37 °C in a humidified incubator.

### Activity assay of rhBMP-2

C2C12 cells were seeded in 24-well plates at an initial density of 5×10^4^ cells/well, and were cultured overnight. Following a 12-h starvation period, cells were induced with different concentrations of rhBMP-2(0, 1, 4, 8, 16, 32 ng/ml) for 72 h. An ALP substrate kit (Bio-Rad, Hercules, CA, USA) was used to determine activity of rhBMP-2.

### Lung metastasis of breast cancer in Balb/c mice

Balb/c mice (4–5 weeks old) were obtained from the Experimental Animal Research Centre of Zhongshan University, and were maintained in its SPF laboratory. Female Balb/c mice (60), 4–6 weeks of age, were randomly divided into three groups (*n*=20). Two groups were intravenously injected with 4T1 cells, and were defined as experimental groups, whereas the un-injected group was set as blank control. Mice in one of the experimental groups were treated daily with 20 *μ*g rhBMP-2 via the tail vein for 21 days; the other two groups were treated with PBS for 21 days. After animals were sacrificed, lung tumor nodules were counted, and lung tissues were fixed in formalin.

### 3D spheroid formation in MCF-7 cells

Matrigel (60–100 *μ*l) was embedded into the bottom of a 24-well plate to form a 0.5–1-mm thick solidified gel layer. MCF-7 cells were dissociated and diluted to a density of 10^3^ cells/ml. Complete medium containing 5% Matrigel was mixed with the cell suspension at a 1:1 ratio and seeded onto a 24-well plate. Cell samples were divided into three groups, which were induced by different concentrations of rhBMP-2 (0, 4, 8 ng/ml). All cell cultures were incubated at 37 °C and 5% CO_2_ incubator for three days, after which the tumor spheres were observed under an inverted microscope. The diameters of 30 randomly chosen tumor spheres were measured for each group.

### Flow cytometry assay

MCF-7 cells were cultured in six-well plates, and were divided into three groups (blank control, 4 ng/ml rhBMP-2-induced and siSmad4 4 ng/ml rhBMP-2-induced). Cells in the siSmad4 4 ng/ml rhBMP-2-induced group were transiently transfected with Smad4 siRNA for 24 h. Both the 4 ng/ml rhBMP-2-induced group and siSmad4 4 ng/ml rhBMP-2-induced groups were then induced with 4 ng/ml rhBMP-2 for 24 h. Cells in each group were incubated with fluorophore-conjugated CD44 or CD24 antibodies for 30 min on ice. They were then washed with HBSS, and fluorescent signals were measured by the FACSCalibur flow cytometer (Becton Dickinson, San Jose, CA, USA).

### Western blot

Proteins were extracted using the Nuclear and Cytoplasmic Extraction Reagents kit (Thermo Fisher Scientific), according to the manufacturer’s protocol. Equal amounts of protein (50 *μ*g) were subjected to sodium dodecyl sulfate–polyacrylamide gel electrophoresis (SDS-PAGE) and western blotting. Membranes were incubated overnight at 4 °C with primary antibodies against Rb (Cell Signaling Technology, Danvers, MA, USA), p-Rb (Ser807/811, Cell Signaling Technology), CD44 (Cell Signaling Technology), p-AKT (Ser473, Cell Signaling Technology), p-ATK (Thr308, Cell Signaling Technology), Histone3 (Cell Signaling Technology), AKT (Cell Signaling Technology) and *β*-actin (Proteintech Group, Rosemont, IL, USA). Membranes were washed in TBS-Tween, and were incubated with anti-mouse secondary antibodies at a dilution of 1:8000 for 1 h. Immunoreactive bands were visualized using an enhanced chemiluminescence reaction kit (Thermo Fisher Scientific), and were exposed on X-ray films (Kodak, Rochester, NY, USA).

### Immunocytochemistry

MCF-7 cells grown on cover slips were washed twice with PBS, and then fixed with 4% paraformaldehyde for 15 min. They were then incubated with 200 *μ*l of 100 nM anti-CD44 (FITC) antibody (Sigma-Aldrich, St Louis, MO, USA) in the dark for 1 h. Cells were subsequently washed three times in PBS to remove any unbound antibodies. Fluorescent images were obtained with a Carl Zeiss LSM510 Meta Duo Scan laser scanning confocal microscope.

### Wound-healing assay

Breast cancer cells were seeded onto 12-well plates. When the cells achieved 80% confluence, they were serum-starved for 12 h in DMEM supplemented with 1% FBS (1 mM thymidine). A wound was made across the cell monolayer using the tip of a 200-*μ*l pipette. The adherent monolayer was washed twice with DMEM at 37 °C to remove the non-adherent cells. Cells were then incubated with different concentrations (0, 4 ng/ml) of rhBMP-2 for another 24 h. The open wound surface area and the number of the cells in the area were photographed by an inverted phase-contrast microscope (Olympus, Tokyo, Japan) and quantified with ImagePro Plus software. All experiments were repeated three times.

### Transwell migration experiment

Cell migration and invasion were examined by a transwell assay using QCMTM 96-well plates (Chemicon, Temecula, CA, USA). After trypsinization, 100 *μ*l 5×10^5^ cells/ml were added to the upper surface of a 96-well plate, 150 *μ*l of different concentrations (0, 4 ng/ml) of rhBMP-2 was plated and used as a chemoattractant in the bottom well. After 24 h of incubation, cells on the upper surface were removed by PBS and 150 *μ*l dissociation buffer was added into each well, and further incubated at 37 °C for 30 min. CyQuant GR staining reagents (50 *μ*l) were added to the mixture for 15 min at room temperature. Samples were transferred to a new low fluorescence background 96-well plate, which was read using 480/520 nm transmitted light.

### Ubiquitination inhibition assay

MCF-7 cells were grown in complete DMEM with 10% FBS for 24 h, and were serum-starved for 12 h with 1% FBS. The ubiquitin-activating enzyme E1 inhibitor, PYR-41 (5 *μ*M, Selleckchem, Houston, TX, USA) was added to MCF-7 cells for 30 min, followed by rhBMP-2 (4 ng/ml) induction for 24 h with 5% FBS. Proteins were extracted using the Nuclear and Cytoplasmic Extraction Reagents (Thermo Fisher Scientific). Equal amounts of protein (40 *μ*g) were subjected to SDS-PAGE, and protein levels of Rb and p-Rb (Ser807/811) were detected by western blots.

### Statistics

Data are presented as mean±S.D.s derived from multiple experiments, as indicated. Differences were assessed by the unpaired-Student’s *t*-test. P<0.05 was considered to be statistically significant (*P<0.05; **P<0.01; ***P<0.005).

### Study approval

All animal procedures were approved by the Center for Animal at Jinan University in accordance with the NIH Guide for the Care and Use of Laboratory Animals (National Academies Press 2011) and the Animal Welfare Act. The immunohistochemistry study used breast carcinoma tissues was approved by the ethics committee of the First Affiliated Hospital of Jinan University, and was carried out according to the IRB-approved protocol. Each patient was properly informed and informed consents were obtained from all patients prior to the study.
