# Supplementary material for: BMP-2 induces EMT and breast cancer stemness through Rb and CD44
Source: Cell Death Discov. 2017 Jul 17;3:17039–. doi: 10.1038/cddiscovery.2017.39 (PMC5511860; doi:10.1038/cddiscovery.2017.39)
Supplement: Supplementary Information [file cddiscovery201739-s1.docx]

**Supplementary materials**

**Supplementary methods**

**Immunohistochemistry.** A total of 40 breast carcinoma tissue samples were collected from First Affiliated Hospital of Jinan University. Each patient was properly informed and informed consents were obtained from all patients prior to the study. The study was approval by the ethics committee of the First Affiliated Hospital of Jinan University. The tissues were fixed in formalin and embedded in paraffin. Tissue slides were deparaffinized with xylene and rehydrated through a graded series of alcohol. The endogenous peroxidase activity was blocked by incubation in 0.2% Triton X-100 for 15 min. Antigen retrieval was done in citrate buffer at a boiling temperature for 10 min. Following rinsing in phosphate-buffered saline(PBS), the slides were blocked with 5% bovine serum albumin (BSA) for 30 min and then were incubated in a 1:200 dilution of anti-mouse BMP-2(Boster, China), Rb, CD44 overnight at 4°C. Incubated secondary antibody for 1h at room temperature. The slides were visualized with 3,3'-diaminobenzidine tetrahydrochloride (DAB)(Dingguo, China) and counterstained in hematoxylin, dehydrated in alcohols, cleared in xylene and mounted. The widely accepted German semi-quantitative scoring system in considering the staining intensity and area extent was used. Each specimen was assigned a score according to the intensity (no staining=0, weak staining=1, moderate staining=2, strong staining=3) and the extent of stained cells (0%=0, 1-25%=1, 26-50%=2, 51-75%=3, 76-100%=4). The final immunoreactive score was determined by multiplying the intensity score with the extent of score of stained cells, ranging from 0 to 12.

**pcDNA3.1-Rb plasmid construction.** Endo-free plasmid mini kit I (Omega, USA) was used to obtain pcDNA3.1-Rb plasmid and pcDNA3.1-Rb vector plasmid. We used pcDNA3.1-Rb plasmid as template, with downstream primer HA tag, PCR to amplify 3’ end with full-length HA tag of Rb1 cDNA fragment. Primers included the following: Rb, forward: 5’-ATATGGATCCGCCGCCACCATGCCGCCCAAAACCCCC-3’; reverse: 5’- GCGCAAGCTTTCACGCATAGTCAGGAACATCGTATGGGTATTTCTCTTCCTTGTTT-3’. CD44, forward: 5'-GACACATATTGCTTCAATGCTTCAGC-3'; reverse: 5'-GATGCCAAGATGATCAGCCATTCTGGAA-3'. MCF-7 cells were seeded into six-well plates, grown to 60% confluence and then transfected with different ratio of transfection reagent (μL) and plasmid DNA (μg) (1:0, 1:1, 2:1, 3:1, 4:1) at 37°C for 24h using X-treme GENE HP DNA transfection reagent (Roche, USA). The cells were allocated to four groups: non-transfection group, vector-transfection group, reagent-transfection group and Rb transfection. Wound healing assay and transwell migration experiment were performed to detect the variation of the MCF-7 cells migration. The protein levels were analyzed by Western blotting.

**CD44 promoter Luciferase assays.** The human CD44 promoter (3135bp fragment upstream of translation initiation Site，cited in UCSC) was whole-gene synthesized and loaded into the pGL3-basic reporter vector to form the CD44 reporter plasmid. MCF-7 cells were transfected with pGL3-basic control plasmid, CD44 reporter plasmid, Renilla luciferase expression construct, siNC and siSamd4 respectively and incubated for 24 hours. And then BMP-2(4ng/ul) was added to the cells. 48 hours after transfection, cells were lysed and tested by Dual Luciferase Assay (Promega), according to the manufacturer’s instructions.

**PCR-array.** We used Human Tumor Metastasis RT² *Profiler*™ PCR Array (QIAGEN) to detect the expression changes of 84 genes known to be involved in tumor metastasis (Supplementary Table. S2). MCF-7 cells were incubated with different concentrations (0, 4ng/mL) of rhBMP-2 for 24 h, and then, total RNA extraction and synthesis of cDNA were carried out according to previous methods. RNA purification was carried out by using RNeasy® MinElute™ purification kit(Qiagen). Quantitative real-time reverse transcription (RT)-PCR was performed with the 2×Super Array PCR master Mix in ABI PRISM7900 system (Applied Bio systems).The above process was repeated for three times. Data analysis was carried out by using Opticon monitor 3 software. Expressions of genes were calculated according to the following formula, △Ct (group 1) = average Ct – average of HK genes’ Ct for group 1 array. Then in terms of 2^-△△ Ct^ (△△Ct = △Ct (group 2) - △Ct (group 1)), counting the differences of expression of corresponding gene.

**RNA Interference.** SiRNA duplexes against human Rb, CD44, Smad4 and control (scrambled) siRNA were synthesized by Shanghai GenePharma. MCF-7 cells were seeded into six-well plates, grown to 30–50% confluence and then transfected with 100pmol Stealth RNAi for 24h using Lipofectamine 2000 (Invitrogen，USA) according to the manufacturer's instructions. The cells were allocated to three groups: si-negative control group, si-Rb/si-CD44/si-smad group and reagent-transfection lipo2000 control group. The protein levels were analyzed by western blotting. Each group was detected with wound healing assay.

**Supplementary Table. S1 Autocrine of BMP-2 in MCF-7 cells.**

| Methods of proteins extraction | Concentration of BMP-2 (pg/mL) | Concentration of total protein (mg/mL) | Expression of BMP-2 (pg/mg) |
| --- | --- | --- | --- |
| NaCl High Salt Buffer | 100.08 | 0.2 | 499.59 |
| Lysis Buffer | 700.08 | 1.964 | 356.46 |

**Supplementary Table. S2 Human Tumor Metastasis RT² *Profiler*™ PCR Array (QIAGEN).**

| **classify** | **Genes** |
| --- | --- |
| ***Cell to Cell Adhesion*** | APC, CD44, CDH1 (E-cadherin), CDH11, CDH6, FAT1, FXYD5, ITGA7, PNN, SYK, VEGFA |
| ***Transmembrane Receptors*** | CD44, ITGA7, ITGB3, RPSA (LAMR1) |
| ***Other Genes Related to Adhesion*** | CTNNA1, FN1, MCAM, MGAT5 ,MTSS1 |
| ***Matrix Metalloproteinases*** | MMP10, MMP11, MMP13, MMP2, MMP3, MMP7, MMP9 |
| ***MMP Inhibitors*** | TIMP2, TIMP3, TIMP4 |
| ***Other ECM Proteins*** | COL4A2 (collagen α2(IV)), HPSE (heparanase) |
| ***Regulation of the Cell Cycle*** | HRAS, IL1B, KRAS, TGFB1 (TGF-β1), VEGFA |
| ***Negative Regulation of the Cell Cycle*** | APC, BRMS1 (BrMS1), CDKN2A, MTSS1, NF2, NME1, NME2, PTEN, RB1, TP53 |
| ***Cell Cycle Arrest and Checkpoin*** | CDKN2A, MYC (c-myc), RB1, TP53 |
| ***Negative Regulation of Cell Proliferation*** | CDKN2A, CTBP1, GNRH1, IL1B, MDM2, NF2, NME1, NME2, SSTR2 |
| ***Positive Regulation of Cell Proliferation*** | IGF1, IL18, TSHR, VEGFA |
| ***Growth Factors and Hormones*** | GNRH1, HGF (Scatter Factor), IGF1, TGFB1 (TGF-Β1), VEGFA |
| ***Cytokines and Chemokines*** | CCL7, CXCL12, IL18, IL1B, TNFSF10 |
| ***Receptors*** | CXCR4, EPHB2, FGFR4, FLT4, KISS1R, CXCR2, MET, NR4A3, PLAUR (uPAR), RORB, SSTR2, TSHR |
| ***Other Genes Related to Growth*** | DENR, EWSR1, HRAS (c-hRas), MYC (c-myc), SET, SRC (c-src), SYK, TRPM1 |
| ***Induction of Apoptosis*** | HTATIP2, IL18, TIMP3, TNFSF10, TP53 |
| ***Anti-apoptosis*** | HTATIP2, TGFB1 |
| ***Other Genes Related to Apoptosis*** | CXCR4, IL1B |
| ***Transcription Factors*** | ETV4, HTATIP2, MTA1, MYC (c-myc), MYCL1, NME2, NR4A3, RB1, RORB, SMAD4, TCF20, TP53 |
| ***Transcription regulators*** | CHD4, EWSR1, SMAD2 |
| ***Other Genes Related to Metastasis*** | CST7, CTSK, CTSL1 (cathepsin L), CD82 (KAI1), KISS1 (KiSS-1), METAP2, NME4 |

**Supplementary Table. S3 Differential expressions of the 84 metastasis relative genes between rhBMP-2 induced MCF-7 cells and the blank control.**

| **Gene**  **Symbol** | **AVG ΔCt (Ct(GOI) - Ave Ct (HKG))** | | **2^^-ΔCt^** | | **Fold Difference** | **T-TEST** |
| --- | --- | --- | --- | --- | --- | --- |
|  | **BMP2** | **CON** | **BMP2** | **CON** | **BMP2 /CON** | **p value** |
| APC | 11.12594425 | 11.330684 | 0.000447463 | 0.000388261 | 1.152478431 | 0.312550506 |
| BRMS1 | 4.09292825 | 4.462917 | 0.058601108 | 0.045344865 | 1.292342753 | 0.037248183 |
| CCL7 | 17.06178758 | 17.03851867 | 7.30954E-06 | 7.42839E-06 | 0.984000588 | 0.819984595 |
| CD44 | 1.095 | 2.455743333 | 0.468136124 | 0.182283599 | 2.568174679 | 0.000243289 |
| CDH1 | 11.05259958 | 9.465576 | 0.0004708 | 0.001414418 | 0.332857462 | 0.000179114 |
| CDH11 | -0.99516442 | -1.297869667 | 1.99330768 | 2.458655609 | 0.810730739 | 0.05396466 |
| CDH6 | 17.06178758 | 17.03851867 | 7.30954E-06 | 7.42839E-06 | 0.984000588 | 0.819984595 |
| CDKN2A | 17.06178758 | 17.03851867 | 7.30954E-06 | 7.42839E-06 | 0.984000588 | 0.819984595 |
| CHD4 | 1.59945125 | 1.884424 | 0.330002475 | 0.270851878 | 1.21838725 | 0.043336911 |
| COL4A2 | 8.029441583 | 7.972892 | 0.003827342 | 0.003980342 | 0.961561086 | 0.72994591 |
| CST7 | 7.119843583 | 7.020609 | 0.007189745 | 0.007701691 | 0.93352814 | 0.59295903 |
| CTBP1 | 3.352210583 | 3.050082333 | 0.097922854 | 0.120735151 | 0.811055053 | 0.153045794 |
| CTNNA1 | 0.361316583 | 0.322804 | 0.778453849 | 0.799514442 | 0.97365827 | 0.857942174 |
| CTSK | 4.338753917 | 4.640653667 | 0.049420249 | 0.040088891 | 1.23276666 | 0.012036878 |
| CTSL1 | 17.06178758 | 17.03851867 | 7.30954E-06 | 7.42839E-06 | 0.984000588 | 0.819984595 |
| CXCL12 | 6.188738583 | 5.769816 | 0.013708946 | 0.018327883 | 0.747983016 | 0.014371219 |
| CXCR4 | 7.21561225 | 6.146203667 | 0.006727974 | 0.014119143 | 0.476514301 | 0.011862136 |
| DENR | 4.718767583 | 3.600200667 | 0.037976017 | 0.082457774 | 0.460551081 | 0.020125342 |
| EPHB2 | 8.13169825 | 8.056163 | 0.003565455 | 0.003757105 | 0.948989974 | 0.755066737 |
| ETV4 | 12.61736125 | 12.040641 | 0.000159146 | 0.000237359 | 0.670486296 | 0.038470564 |
| EWSR1 | 0.745831583 | 0.540285333 | 0.596324046 | 0.687634897 | 0.867210272 | 0.173228852 |
| FAT | 5.672691917 | 6.875935 | 0.01960422 | 0.008514072 | 2.302566915 | 0.001328396 |
| FGFR4 | 8.921909583 | 8.655436667 | 0.002061758 | 0.002480013 | 0.831349537 | 0.097496865 |
| FLT4 | 7.33167625 | 7.123154 | 0.006207912 | 0.007173267 | 0.865423229 | 0.414508016 |
| FN1 | 5.564739917 | 6.655989667 | 0.021127415 | 0.00991625 | 2.130585208 | 5.25162E-05 |
| FXYD5 | 8.48715925 | 8.930455333 | 0.00278683 | 0.002049581 | 1.359707265 | 0.197853517 |
| GNRH1 | 8.56717525 | 8.293518333 | 0.002636473 | 0.003187148 | 0.827220064 | 0.358764135 |
| KISS1R | 17.06178758 | 16.60579733 | 7.30954E-06 | 1.00267E-05 | 0.729009616 | 0.316239574 |
| HGF | 16.30241358 | 17.03851867 | 1.23733E-05 | 7.42839E-06 | 1.665672862 | 0.417688147 |
| HPSE | 10.09075192 | 9.326994667 | 0.000917025 | 0.001557022 | 0.588960486 | 0.027051874 |
| HRAS | 2.85759625 | 2.446384667 | 0.137967824 | 0.183469904 | 0.751991582 | 0.061928393 |
| HTATIP2 | 3.369897583 | 3.351752667 | 0.096729679 | 0.09795394 | 0.987501663 | 0.957863469 |
| IGF1 | 16.79545758 | 16.74353333 | 8.79151E-06 | 9.11369E-06 | 0.964648833 | 0.921049506 |
| IL18 | 6.89068825 | 7.505339667 | 0.008427449 | 0.005503863 | 1.531187987 | 0.00103465 |
| IL1B | 11.56519292 | 9.700673 | 0.000330012 | 0.001201729 | 0.274614571 | 0.001185477 |
| IL8RB | 17.06178758 | 16.91914267 | 7.30954E-06 | 8.0692E-06 | 0.90585691 | 0.304004681 |
| ITGA7 | 15.70005425 | 16.75130967 | 1.87851E-05 | 9.0647E-06 | 2.072332383 | 0.149007538 |
| ITGB3 | 12.52618492 | 11.439525 | 0.000169528 | 0.000360048 | 0.470850212 | 0.024069851 |
| CD82 | 8.20695525 | 7.319684 | 0.003384233 | 0.006259729 | 0.540635724 | 0.000182173 |
| KISS1 | 10.75686292 | 11.57903533 | 0.000577912 | 0.000326861 | 1.768066351 | 0.050798654 |
| KRAS | 4.55979425 | 4.554938667 | 0.042399932 | 0.042542875 | 0.996640023 | 0.976764538 |
| RPSA | -0.58992808 | -1.306252 | 1.505171714 | 2.472982448 | 0.60864634 | 0.006705955 |
| MCAM | 12.39943392 | 12.00036167 | 0.000185097 | 0.000244079 | 0.758345794 | 0.270927587 |
| MDM2 | 2.561740583 | 2.43601 | 0.169371075 | 0.184794023 | 0.916539788 | 0.480966568 |
| MET | 10.28668558 | 9.952709333 | 0.00080057 | 0.001009104 | 0.793346903 | 0.4811029 |
| METAP2 | 10.71992258 | 9.993302 | 0.0005929 | 0.000981107 | 0.604317831 | 0.051882034 |
| MGAT5 | 5.855928917 | 5.823872 | 0.017265921 | 0.017653866 | 0.978024888 | 0.853049024 |
| MMP10 | 14.99741258 | 15.36121033 | 3.05724E-05 | 2.37583E-05 | 1.286808838 | 0.821865511 |
| MMP11 | 6.98075925 | 8.456945667 | 0.007917391 | 0.002845809 | 2.78212341 | 0.004573616 |
| MMP13 | 17.06178758 | 17.03851867 | 7.30954E-06 | 7.42839E-06 | 0.984000588 | 0.819984595 |
| MMP2 | 17.06178758 | 16.96010633 | 7.30954E-06 | 7.84331E-06 | 0.931946311 | 0.331747571 |
| MMP3 | 17.06178758 | 17.03851867 | 7.30954E-06 | 7.42839E-06 | 0.984000588 | 0.819984595 |
| MMP7 | 17.06178758 | 17.03851867 | 7.30954E-06 | 7.42839E-06 | 0.984000588 | 0.819984595 |
| MMP9 | 6.842836583 | 7.230402333 | 0.00871166 | 0.006659353 | 1.30818425 | 0.122600347 |
| MTA1 | 2.19321425 | 2.222428667 | 0.218663716 | 0.214280331 | 1.020456311 | 0.787222701 |
| MTSS1 | 3.816783917 | 2.969033667 | 0.070963259 | 0.12771203 | 0.555650547 | 0.000328805 |
| MYC | 3.94476025 | 3.861317667 | 0.064939485 | 0.068806198 | 0.943802839 | 0.780208517 |
| MYCL1 | 10.36327158 | 9.411569 | 0.000759179 | 0.00146837 | 0.517021943 | 0.100658112 |
| NF2 | 7.14626225 | 7.313930333 | 0.007059285 | 0.006284744 | 1.123241452 | 0.428637327 |
| NME1 | 0.78680325 | 0.641518667 | 0.579627018 | 0.641037799 | 0.904200999 | 0.151800792 |
| NME2 | -0.72264942 | -1.071862333 | 1.650209758 | 2.102145215 | 0.785012256 | 0.017243951 |
| NME4 | 0.846401583 | 1.150098333 | 0.556170225 | 0.450594518 | 1.234303133 | 0.1376649 |
| NR4A3 | 5.69653625 | 5.77137 | 0.019282872 | 0.018308152 | 1.053239658 | 0.84361083 |
| PLAUR | 7.21649925 | 7.644839333 | 0.006723838 | 0.004996594 | 1.345684386 | 0.095582071 |
| PNN | 2.53708225 | 2.145077333 | 0.172290821 | 0.226082725 | 0.762069818 | 0.06043725 |
| PTEN | 2.27511825 | 2.580587667 | 0.206595645 | 0.167172834 | 1.235820677 | 0.096527076 |
| RB1 | 5.73025525 | 3.678133 | 0.018837414 | 0.078121692 | 0.241129113 | 0.000658136 |
| RORB | 16.84461058 | 17.03851867 | 8.49703E-06 | 7.42839E-06 | 1.143858094 | 0.12235488 |
| SET | 0.42568725 | 0.131064667 | 0.744484001 | 0.913157318 | 0.815285588 | 0.240012051 |
| SMAD2 | 6.695602917 | 6.584946 | 0.009647675 | 0.010416786 | 0.926166245 | 0.321678225 |
| SMAD4 | 5.238427583 | 4.534288 | 0.026489647 | 0.043156211 | 0.613808452 | 0.06907737 |
| SRC | 7.852962917 | 8.374799667 | 0.004325363 | 0.003012549 | 1.435782034 | 0.124463116 |
| SSTR2 | 10.59468425 | 9.961516333 | 0.000646669 | 0.001002963 | 0.644759077 | 0.111338635 |
| SYK | 7.048732917 | 6.149325667 | 0.007553008 | 0.014088622 | 0.536106953 | 0.009405004 |
| TCF20 | 4.55627225 | 4.441415333 | 0.042503568 | 0.046025738 | 0.923473894 | 0.714389707 |
| TGFB1 | 3.87262325 | 4.481162333 | 0.06826911 | 0.044775013 | 1.524714455 | 0.000105605 |
| TIMP2 | 1.609723583 | 1.942342667 | 0.327661124 | 0.260193591 | 1.259297442 | 0.26318673 |
| TIMP3 | 14.38889425 | 15.62375533 | 4.66134E-05 | 1.98053E-05 | 2.353586837 | 0.169591109 |
| TIMP4 | 12.36297525 | 11.32990067 | 0.000189834 | 0.000388472 | 0.48866762 | 0.011978177 |
| TNFSF10 | 7.385125917 | 6.907787333 | 0.005982126 | 0.008328155 | 0.718301492 | 0.033794703 |
| TP53 | 0.64479725 | 0.544627 | 0.639582668 | 0.685568629 | 0.932922892 | 0.420084988 |
| TRPM1 | 16.38491692 | 16.484884 | 1.16855E-05 | 1.09032E-05 | 1.071749009 | 0.873894526 |
| TSHR | 15.75620792 | 15.99608167 | 1.80679E-05 | 1.53003E-05 | 1.180889317 | 0.794468598 |
| VEGFA | 3.687049583 | 2.936842 | 0.077640349 | 0.130593772 | 0.594518009 | 0.009891522 |

**Supplementary Table. S4 The human CD44 promoter (3135bp fragment upstream of translation initiation Site，cited from UCSC) for construction of the CD44 reporter plasmid.**

| **3135bp fragment upstream of CD44 translation initiation Site (SBEs are highlight by yellow).**  **The first exon of CD44 also show in red letters.** |
| --- |
| 5’-GAGGGGTCTCTAAGGAACCTCCAGGTTCCATGAAACACAGTAAGACAGACATTGTTCCTAGTGACTCCCCACGTCACTTCACTTTCTTGCTCCAAGAAAAGGAAGCTGAAATCCCTGGAGGTTGCATGAGGTGAAAAAAGCTGCTCCTTGTCCTGTATTCTTGTGCATGGAACCTACCAGGGCTGGTTTCCTCACATGAGTAGGGACTGAAGCCTTATCTGGGAGCCAGTTTGCCCCCAGTTGTTTCAAGAAATAAAGTGTCCTCTTGGTCACAGACAGCAGCAGTAACAACTTTTTCCTGCGGACTGGCAGCATTTTCCAAGCACCTGCTCTTCAGGTGGGAGTTACGGGTCACTGTCTTCATTCCAGACGGGCGGCTCTTTGAGTCATGGCATGCCAGCTTCTGAGTGGCTGAGGACTAGATCCAGACAAGGATTTTTCAGTCCCAAACACTCTTCCTGAAAATGCAAGAGGAGTAGTCTTTAGTCAAACAGAATCTCCACAACTGAAAAATTTATACCGGACTAGACAGTGGTAGTGGTTGCACAATATTGTTAAAGTACTACACGTCACTGCTATAAACACTTTTAAATGGGTGAAATGGTAAATTTTATGTTATGCATATTTTAACATAACAATGATAATAATTTTAACTAAGTACGTTGCCTCCTTATTTCCTACAGGGTCAAGTTCAAACTCCCTGGCATAGCTTACACCTTGTAGTCCCCATACCTGTAACCTCATGTCTGCTATCTTCCCCCAGCCCCTTCATCTGATCTATGTTCAGATCAGCTCACATTGCTCTCTATTCTCTGGACAAGCCATGTTCTTTCACTTTTCTATGCTCAAACACAATTTTGCTTTTAGTAATGATAGCAAACACTTATTTAAAGTGCTTACTGCATGCATATGTAGACATAAATCTACGGCTTAATACATGCAGGGCTTTTGAGTCATTTAATCCTCACCAATTCTATGAAGTAATAGAATTGTAATAGTAATAATAGATACTATTATTAAGCCCATTTTATACTTGAGCAAATTGAGCTAATTGCCTTAGGCAAGGTCACACAACTAAGAAGCAGCCATAATAGGGTTTGGATCTAGGTGTTCTAGCTCCTGAATCCATGCTGTTCGTCACTACACTGTACTGCCTGTGGATGACTTACTTGTCCCTGTAGTTTCATCTGAAGAATTCCTCCTCCTTTTCCTTTGAGGCCTGCCTCAAATATCACTTCCCCTGTGAAGACTGCCTGGTGTTCTCCAGGAGAGAGTGTGACTCCCTTCTCTAGGAATGGTAGCACCCCAAACACACACATTTTGCAGCATATTTCACCTTGCATGGTAATGGCCTGCTTGTGAGTTTTATTCCGTACCAGAGGGTGAGGGCTCTGAAGATAGCGCCAGGTCTTATTTACCTCGATACCCCACAACACTCATTACATGTCTGATGAATGAATGCATAGGGGGATGGCTGGGTGCATTTCTCTCAACTTTTCAATTTCTTGAAATAAATAACAAAATCTTACCTTCCCTCAGAAGTCCTGGCATGGTTCCTTTCATCTTGCCACAGCCACTGATAATCACTTTCATTTTCTGTGTAACTCACCAGGCAAGAAGTCCATGCAGATTTACTTTTAGTAGTTCACATGACAAATAAATACTGCGTTTGATTTCCAAACATTAAACCATAGTATATTATAGATAGATATAGAGTTATCATTCAAAGTATGATATTTCAATCTCAAAAGGCTTCCCCTGAAGAATATTACAAACTCTTCCTCTCTTTAAGATCTGCTGGGTAGGAAAGATGGGAGAAAATGAATTAATGTTTACACAGAAAGGAGGATAATGGGGGCAAAAATAATAGATGAACGTATGGGTGGATGAGAGAATGGATAAAATGATAGGTGGATATGTTGATCTTGGACAGATGGGAAATGAGTGGATATATCAATAAACAGATATGTGGGTGGATGGGTGGAGAAGAGGATGGTGGATGGTTGTGGTTTTATGAAGAGATGTGAAAAAGGAAGTGTGGAATGATGGATGAGAAGTTGTATGGGAAGATGAATAGAAGAATAGGTGGTTGAATAAATTAAAAGGTGTGTGGTTGGATGAATGAATGAGTGGGATGATAGATGGACCTAAGTGGTTAGTGGATGGACAGGAGGATGGATGGATGTGAGAGCCCCAGAAGGACATAAGGAAAGATGGGTGGATAGATGGATGGGCGGATGGAAGGATATTTAGGAGGATGAATGAGCATGTGTGTGGAGAGAGGTGCCCATTCACACTGGCTTGAACACATGGGTTAGCTGAGCCAAATGCCAGCCCTATGACAGGCCATCAGTAGCTTTCCCTGAGCTGTTCTGCCAAGAAGCTAAAATTCATTCAAGCCATGTGGACTTGTTATTGAGGGGAAAAAGAATGAGCTCTCCCTCTTTCCACTTGGAAGATTCACCAACTCCCCACCCCTCACTCCCCACTGTGGGCACGGAGGCACTGCGCCACCCAGGGCAAGACCTCGCCCTCTCTCCAGCTCCTCTCCCAGGATATCCAACATCCTGTGAAACCCAGAGATCTTGCTCCAGCCGGATTCAGAGAAATTTAGCGGGAAAGGAGAGGCCAAAGGCTGAACCCAATGGTGCAAGGTTTTACGGTTCGGTCATCCTCTGTCCTGACGCCGCGGGGCCAGCGGGAGAAGAAAGCCAGTGCGTCTCTGGGCGCAGGGGCCAGTGGGGCTCGGAGGCACAGGCACCCCGCGACACTCCAGGTTCCCCGACCCACGTCCCTGGCAGCCCCGATTATTTACAGCCTCAGCAGAGCACGGGGCGGGGGCAGAGGGGCCCGCCCGGGAGGGCTGCTACTTCTTAAAACCTCTGCGGGCTGCTTAGTCACAGCCCCCCTTGCTTGGGTGTGTCCTTCGCTCGCTCCCTCCCTCCGTCTTAGGTCACTGTTTTCAACCTCGAATAAAAACTGCAGCCAACTTCCGAGGCAGCCTCATTGCCCAGCGGACCCCAGCCTCTGCCAGGTTCGGTCCGCCATCCTCGTCCCGTCCTCCGCCGGCCCCTGCCCCGCGCCCAGGGATCCTCCAGCTCCTTTCGCCCGCGCCCTCCGTTCGCTCCGGACACCATGGACAAGTTTTGGTGGCACGCAGCCTGGGGACTCTGCCTCGTGCCGCTGAGCCTGGCGCAGATCG -3’ |

**Supplementary Table S5. Classification of the 40 cases of breast tumor specimens.**

| Classification Index  Tumor Type(cases#) | Hormone Receptor Expression (ER/PR) | CerbB-2 |
| --- | --- | --- |
| Luminal A(9) | Positive | Negative |
| Luminal B(17) | Positive | Positive |
| HER-2(10) | Negative | Positive |
| TNBC(4) | Negative | Negative |

**
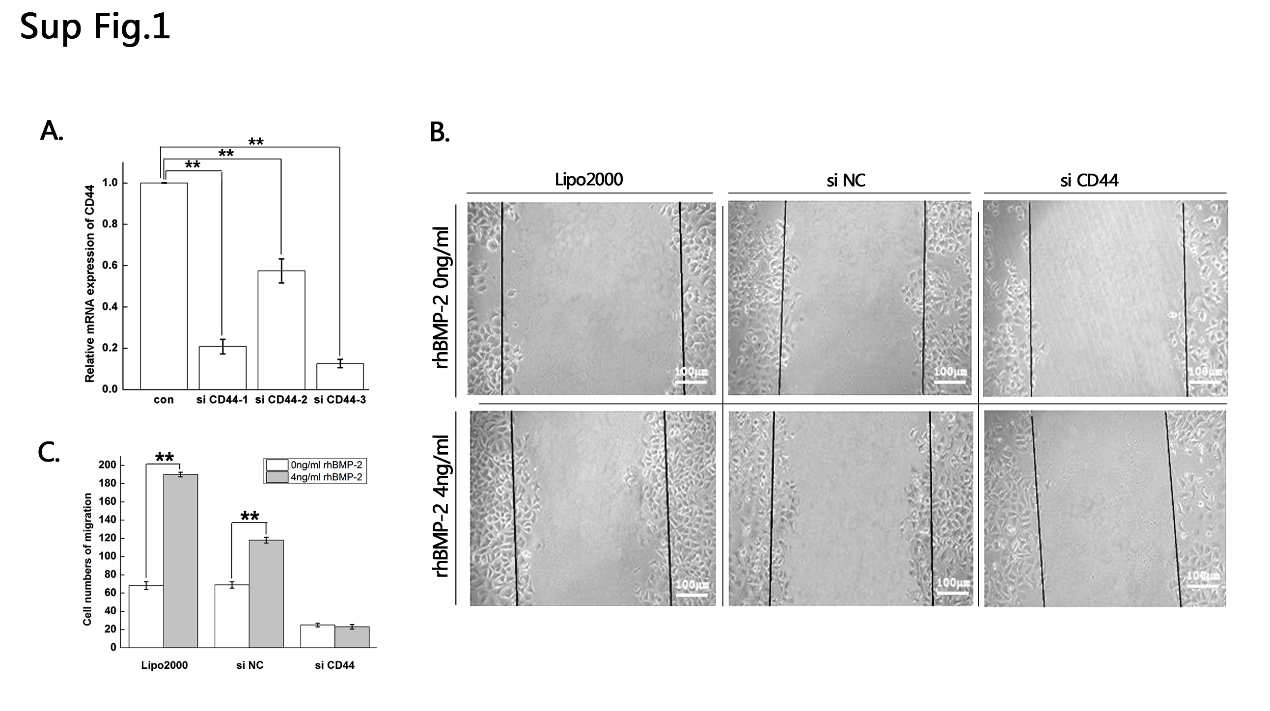
**

**Supplementary Figure 1. Depletion the expression of CD44 protein in MCF-7 cells neutralized the promoting effect of rhBMP-2 on MCF-7 cells migration.**

A. Real Time PCR was applied to evaluate the interference efficient of CD44 siRNA. Data are shown as mean ± SD, (**P < 0.01, n=3). (B,C). Silencing the expression of CD44 protein in MCF-7 cells neutralized the promoting effect of rhBMP-2 on MCF-7 cells migration. Data are shown as mean ± SD, (**P < 0.01, n=3).


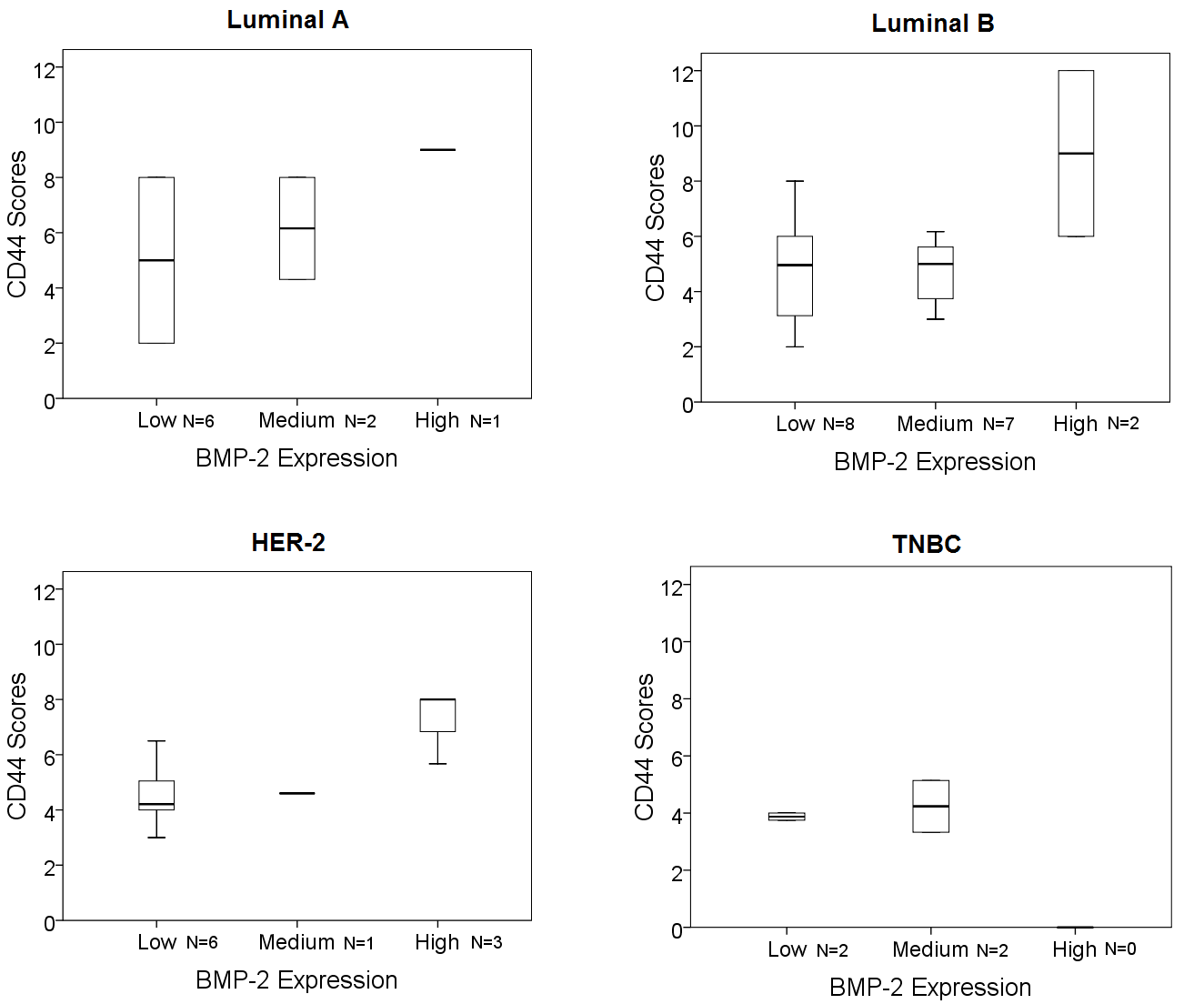


**Supplementary Figure 2. Correlation between BMP-2 and CD44 in each sub-type of breast carcinoma tissues.**


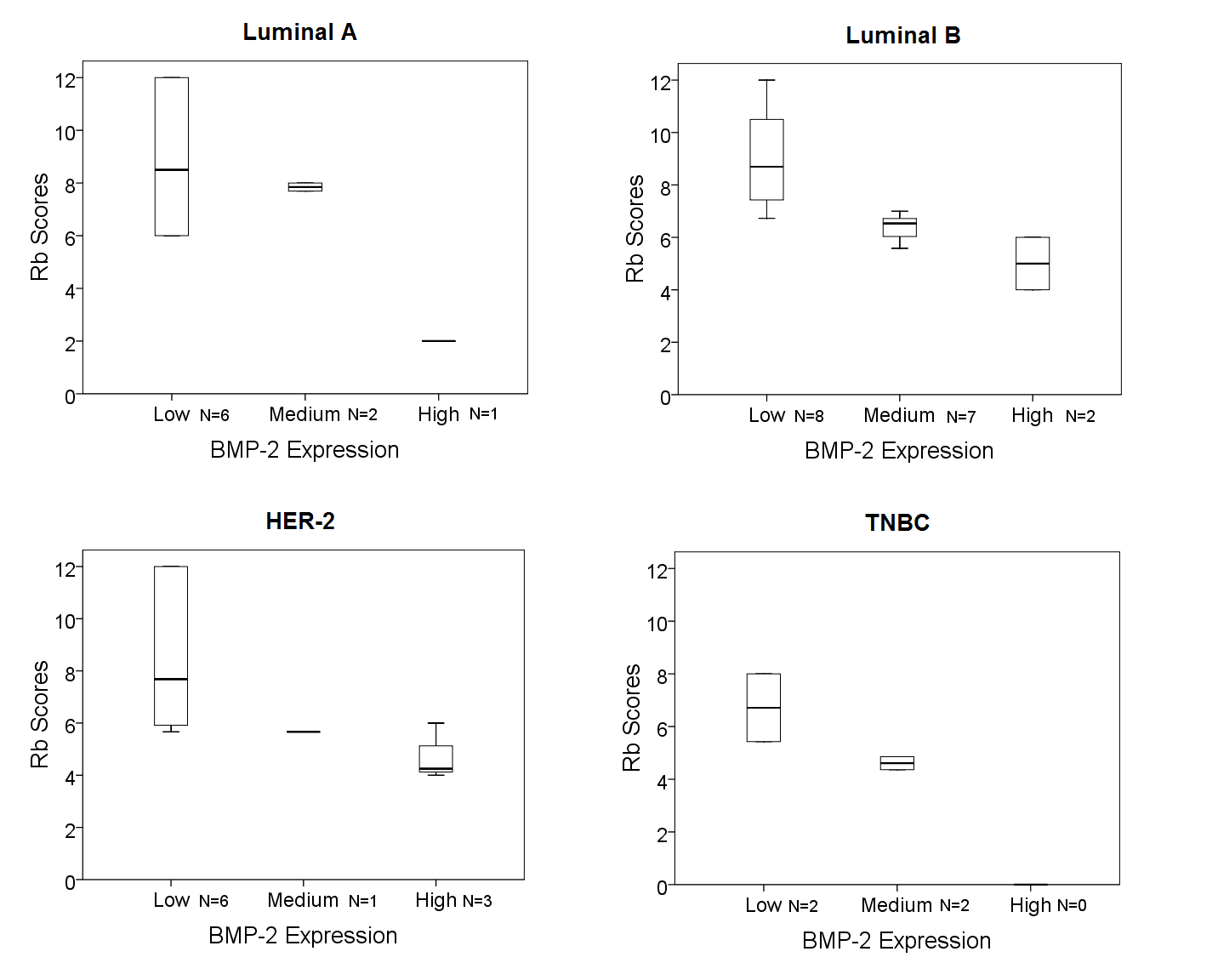


**Supplementary Figure 3. Correlation between BMP-2 and Rb in each sub-type of breast carcinoma tissues.**
